# Acral Melanocytic Lesions Under Conventional, Sub‐Ultraviolet Reflectance, and Ultraviolet‐Induced Fluorescence Dermoscopy: A Comparative Analysis

**DOI:** 10.1111/ijd.70384

**Published:** 2026-03-16

**Authors:** Handan Merve Erol Mart, Ahmet Taha Aydemir, Pawel Pietkiewicz, Bengu Nisa Akay

**Affiliations:** ^1^ Faculty of Medicine, Department of Dermatology Ankara University Ankara Turkey; ^2^ Department of Dermatology Yozgat City Hospital Yozgat Turkey; ^3^ Department of Dermatology Zwierzyniecka Medical Center Poznan Poland

**Keywords:** acral nevus, dermatoscopy, dermoscopy, melanoma, sub‐ultraviolet dermoscopy, ultraviolet dermoscopy

## Abstract

**Background:**

Differentiating benign acral melanocytic lesions (AMLs) from malignant ones remains a diagnostic challenge. While conventional dermoscopy (CD) is widely used, sub‐ultraviolet reflectance dermoscopy (sUVRD) and ultraviolet‐induced fluorescence dermoscopy (UVFD) may enhance feature visualization. This study compared the visibility scoring of CD, sUVRD, and UVFD in AMLs.

**Methods:**

A total of 42 AMLs from 37 patients were retrospectively analyzed, including 22 nevi, 12 melanoma in situ (MIS), and 8 invasive melanomas. Three dermatologists assessed each lesion using CD, sUVRD, and UVFD, scoring 11 features: parallel furrow pattern (PFP), parallel ridge pattern (PRP), fibrillar pattern, dots/clods, radial lines, blue–white structureless area, blotch, scale, ulceration, lesion margins, and eccrine duct openings on a 5‐point scale by consensus.

**Results:**

When all lesions were analyzed collectively, CD provided higher scores than UVFD for PFP, PRP, fibrillar pattern, dots/clods, radial lines, blue–white structureless areas, and blotch, and outperformed sUVRD for PRP and blue–white structureless areas. SUVRD better revealed eccrine duct openings. Scale, ulceration, and lesion margins were consistently well visualized across all three modalities. In subgroup analyses, CD scores were significantly higher than UVFD scores for PFP, fibrillar pattern, and dots/clods in nevi; for PRP and dots/clods in MIS; and for PRP, radial lines, blue–white structureless areas, and blotch in invasive melanoma. SUVRD was superior to CD in demonstrating eccrine duct openings in nevi and to UVFD in MIS.

**Conclusions:**

CD remains superior for visualizing key features of AMLs, while sUVRD and UVFD offer complementary strengths that may enhance overall lesion assessment.

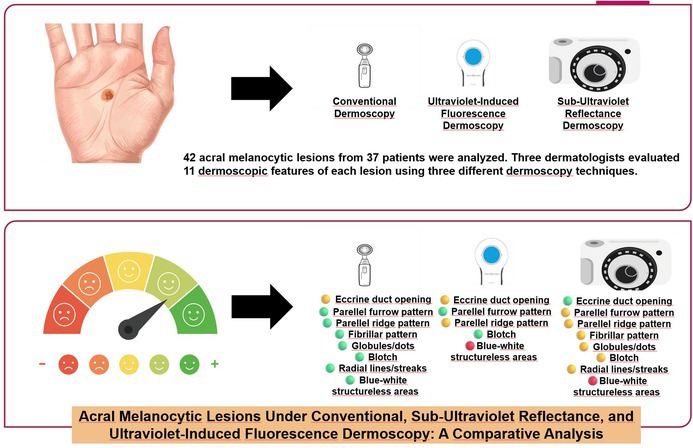

## Graphical abstract

Conventional dermoscopy provided higher scores than ultraviolet‐induced fluorescence dermoscopy for parallel furrow pattern, parallel ridge pattern (PRP), fibrillar pattern, dots/clods, radial lines, blue–white structureless areas and blotch, and outperformed sub‐ultraviolet reflectance dermoscopy (sUVRD) for PRP and blue–white structureless areas. SUVRD better revealed eccrine duct openings. Scale, ulceration, and lesion margins were consistently well visualized across all three modalities.

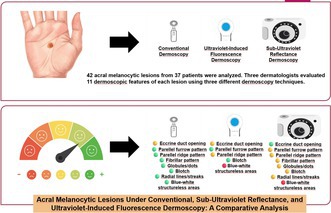

## 1 Introduction

Acral skin, located distal to the Wallace line, is distinguished by its glabrous texture and the presence of dermatoglyphs. This unique anatomical structure leads to distinctive pigment distribution patterns in both benign and malignant lesions. Acral melanoma represents approximately only 3% of all melanomas, yet is often diagnosed at advanced stages, particularly due to late detection and/or misdiagnosis. As delayed diagnosis is a well‐known predictor of poor prognosis, early and accurate identification of this malignant tumor is a major concern [1].

Conventional dermoscopy (CD), employing polarized and nonpolarized light, has significantly improved the evaluation of pigmented skin lesions. Nevertheless, its diagnostic sensitivity may be limited in special anatomical sites such as acral regions, where subtle distinctions between benign and malignant structures—such as the parallel furrow pattern (PFP) typical of nevi versus the parallel ridge pattern (PRP) seen in melanoma—can be difficult to discern. Therefore, novel dermoscopic techniques utilizing ultraviolet and sub‐ultraviolet light have gained interest as complementary tools.

Sub‐ultraviolet reflectance dermoscopy (sUVRD; wavelength 405 nm) is based on reflectance principles: tissues absorb and scatter violet–blue light differently depending on their chromophore composition. This produces high‐contrast grayscale images that can enhance the visualization of melanin distribution and keratin structures. On the other hand, ultraviolet‐induced fluorescence dermoscopy (UVFD; wavelength 365 nm) uses UVA light to induce fluorescence by exciting fluorophores such as keratin, porphyrins, and pteridines, resulting in visible light emission that highlights particular structural components [2]. Recent studies have suggested that sUVRD and UVFD can improve margin assessment, pattern visibility, and detection of subtle pigment changes that are inconspicuous under CD [3, 4, 5, 6, 7, 8].

In this study, we aimed to compare the visibility scoring of CD, sUVRD, and UVFD in acral melanocytic lesions (AMLs), focusing on lesion border delineation, visualization of key dermoscopic patterns, and malignancy‐associated features.

## 2 Materials and Methods

In this retrospective observational study, we analyzed dermatoscopic images of AMLs collected between January 2020 and May 2025 from two participating centers in Turkey and Poland.

For melanomas, only pathology confirmed cases were included, whereas stable clinical course and dermoscopic features consistent with benign patterns were inclusion criterium for nonpathology‐confirmed nevi. Collected data included age, sex, affected site and the diagnosis.

All lesions were imaged using CD and sUVRD, while UVFD was additionally performed in a subset of lesions when available. Among a total of 42 lesions, a complete image set was available for 30 cases, whereas UVFD images were missing in 12 cases. CD and sUVRD were performed with the same DZ‐D100 device (Casio, Japan) in polarized/nonpolarized and 405 nm sub‐UV modes, respectively, whereas UVFD was conducted with a DL5 device (DermLite, USA; 365 nm UV mode). We used noncontact UVFD, while sUVRD was performed in contact mode using 70% isopropyl alcohol as a contact medium.

Each lesion was evaluated by three dermoscopists, and a consensus decision was reached after discussion. A standardized set of 11 dermoscopic features was used and scored with a 5‐point Likert scale (1 = absent, 5 = extraordinary visibility): PFP, PRP, fibrillar pattern, dots/clods (globules), radial lines (streaks), blue–white structureless area (blue–whitish veil), blotch, scale, ulceration, lesion margins, and eccrine duct opening visibility.

Statistical analyses were performed using R software (version 4.3.3; R Foundation for Statistical Computing, Vienna, Austria). Descriptive statistics were presented as mean ± standard deviation for continuous variables and as frequencies (percentages) for categorical variables. The association between gender and acral melanocytic lesion subtype was assessed using Fisher's exact test.

A linear mixed‐effects model was used to assess differences in feature scores across the three dermoscopic modalities. Parameter estimation was performed using restricted maximum likelihood. Degrees of freedom were approximated using the Kenward–Roger method, and pairwise post hoc comparisons were adjusted using the Tukey correction. Missing observations were driven by the unavailability of UVFD imaging in a subset of lesions and were handled inherently within the mixed‐effects modeling framework by including all available data for each modality and feature without listwise deletion. A *p* value < 0.05 was considered statistically significant.

This study was approved by the Clinical Research Ethics Committee of the Ankara University Faculty of Medicine (Approval No: I05‐424‐25), and written informed consent was obtained from all patients.

## 3 Results

### 3.1 Patient and Lesion Characteristics

A total of 42 AMLs from 37 patients were evaluated (mean age: 48.35 ± 25.29 years), including 22 nevi, 12 MIS, and 8 invasive melanomas. The mean age differed significantly between groups: patients with nevi were younger (27.2 ± 15.1 years) compared to those with MIS (66.5 ± 15.1 years) and invasive melanoma (65.0 ± 20.3 years) (*p* < 0.001).

There was an overall female predominance (male/female: 9/28), which was also evident across all diagnostic categories (nevi 14/17, MIS 8/12, invasive melanoma 6/8); however, the association between sex and diagnosis was not statistically significant (*p* = 0.703).

Lesion localization demonstrated a clear predilection for weight‐bearing sites. Nevi, MIS, and invasive melanoma were most commonly located on the sole (72.7%, 41.7%, and 75.0%, respectively). Fitzpatrick skin phototype distribution revealed that skin types II and III were most common across all groups (Tables S1 and S2).

### 3.2 Overall Comparison of Dermoscopic Modalities

Marked differences emerged in the visualization of key dermoscopic parameters across the CD, sUVRD and UVFD modalities. Mean scores differed significantly for PFP, PRP, fibrillar pattern, dots/clods, radial lines, blue–white structureless areas, blotches and eccrine duct openings (*p* = 0.004, *p* < 0.001, *p* = 0.01, *p* = 0.002, *p* = 0.03, *p* < 0.001, *p* = 0.05, and *p* < 0.001, respectively).

Post hoc analyses revealed distinct performance profiles: For the PFP, both CD (4.87 ± 0.52) and sUVRD (4.80 ± 0.56) outperformed UVFD (3.89 ± 1.05) (*p* = 0.008 and *p* = 0.012, respectively).

For the fibrillar pattern, CD (4.67 ± 0.82) surpassed UVFD (3.13 ± 1.48) (*p* = 0.009).

Dots/clods were also more readily identified with CD than UVFD (4.62 ± 0.62 vs. 3.01 ± 1.30, *p* = 0.002).

For radial lines, the mean CD score (4.50 ± 0.76) was higher than UVFD (3.11 ± 0.84) (*p* = 0.024). For blotches, the mean scores of CD (4.89 ± 0.33) and sUVRD (4.44 ± 0.73) were higher than UVFD (3.38 ± 1.51) (*p* = 0.004 and *p* = 0.038, respectively).

CD demonstrated a clear visibility advantage for melanoma‐specific structures, particularly the PRP, with CD scoring significantly higher (4.84 ± 0.38) than sUVRD (4.00 ± 0.94) and UVFD (3.07 ± 1.14) (*p* = 0.011 and *p* < 0.001, respectively), while the mean sUVRD score was higher than UVFD (*p* = 0.009).

For blue–white structureless areas, a hallmark of melanoma progression, CD offered markedly superior visualization (4.86 ± 0.38) compared with sUVRD (2.14 ± 1.46) and UVFD (2.00 ± 1.00) (both *p* = 0.002).

sUVRD excelled in visualizing eccrine duct openings, outperforming both CD and UVFD (3.83 ± 1.32 vs. 2.86 ± 1.47 and 2.61 ± 1.52, both *p* < 0.001).

Notably, scale, ulceration, and lesion margins were consistently well‐visualized across all three methods.

Table 1 provides the detailed comparison of mean visualization scores across modalities.

**TABLE 1:** Evaluation of dermoscopic features using various imaging devices in acral melanocytic lesions.

Dermoscopic findings	Dermoscopic imaging device			*p*	Post hoc Tukey's test
	CD score Mean (SD)	sUVRD score Mean (SD)	UVFD score Mean (SD)		
Eccrine duct opening	2.86 ± 1.47 (*n* = 42)	3.83 ± 1.32 (*n* = 42)	2.61 ± 1.52 (*n* = 30)	**< 0.001**	sUVRD>CD, UVFD
Borders	4.79 ± 0.47 (*n* = 42)	4.81 ± 0.63 (*n* = 42)	4.60 ± 0.62 (*n* = 30)	0.216	—
Parellel ridge pattern	4.84 ± 0.38 (*n* = 19)	4.00 ± 0.94 (*n* = 19)	3.07 ± 1.14 (*n* = 14)	**< 0.001**	CD>sUVRD>UVFD
Globules/dots	4.62 ± 0.62 (*n* = 16)	3.94 ± 1.48 (*n* = 16)	3.01 ± 1.30 (*n* = 11)	**0.002**	CD>UVFD
Parellel furrow pattern	4.87 ± 0.52 (*n* = 15)	4.80 ± 0.56 (*n* = 15)	3.89 ± 1.05 (*n* = 9)	**0.004**	CD, sUVRD>UVFD
Blotch	4.89 ± 0.33 (*n* = 9)	4.44 ± 0.73 (*n* = 9)	3.38 ± 1.51 (*n* = 6)	**0.005**	CD, sUVRD>UVFD
Radial lines/streak	4.50 ± 0.76 (*n* = 8)	4.00 ± 1.20 (*n* = 8)	3.11 ± 0.84 (*n* = 5)	**0.03**	CD>UVFD
Blue–white structureless areas	4.86 ± 0.38 (*n* = 7)	2.14 ± 1.46 (*n* = 7)	2.00 ± 1.00 (*n* = 5)	**< 0.001**	CD>sUVRD, UVFD
Ulceration	4.71 ± 0.76 (*n* = 7)	4.29 ± 1.11 (*n* = 7)	4.36 ± 0.82 (*n* = 6)	0.594	—
Fibrillar pattern	4.67 ± 0.82 (*n* = 6)	4.17 ± 0.98 (*n* = 6)	3.13 ± 1.48 (*n* = 5)	**0.01**	CD>UVFD
Scale	4.80 ± 0.45 (*n* = 5)	4.80 ± 0.45 (*n* = 5)	4.27 ± 0.96 (*n* = 4)	0.309	—

*Note:* Dermoscopic findings that could not be evaluated on any device were excluded from *n*. Bold values indicate statistically significant associations (*p* 〈 0.05).

Abbreviations: CD, conventional dermoscopy; SD, standard deviation; sUVRD, sub‐ultraviolet reflectance dermoscopy; UVFD, ultraviolet‐induced fluorescence dermoscopy.

### 3.3 Subgroup Analyses by Diagnosis

#### 3.3.1 Nevi

CD achieved significantly higher scores than UVFD for the PFP, fibrillar pattern, and dots/clods (*p* = 0.027, *p* = 0.009, and *p* = 0.016, respectively).

**FIGURE 1:**
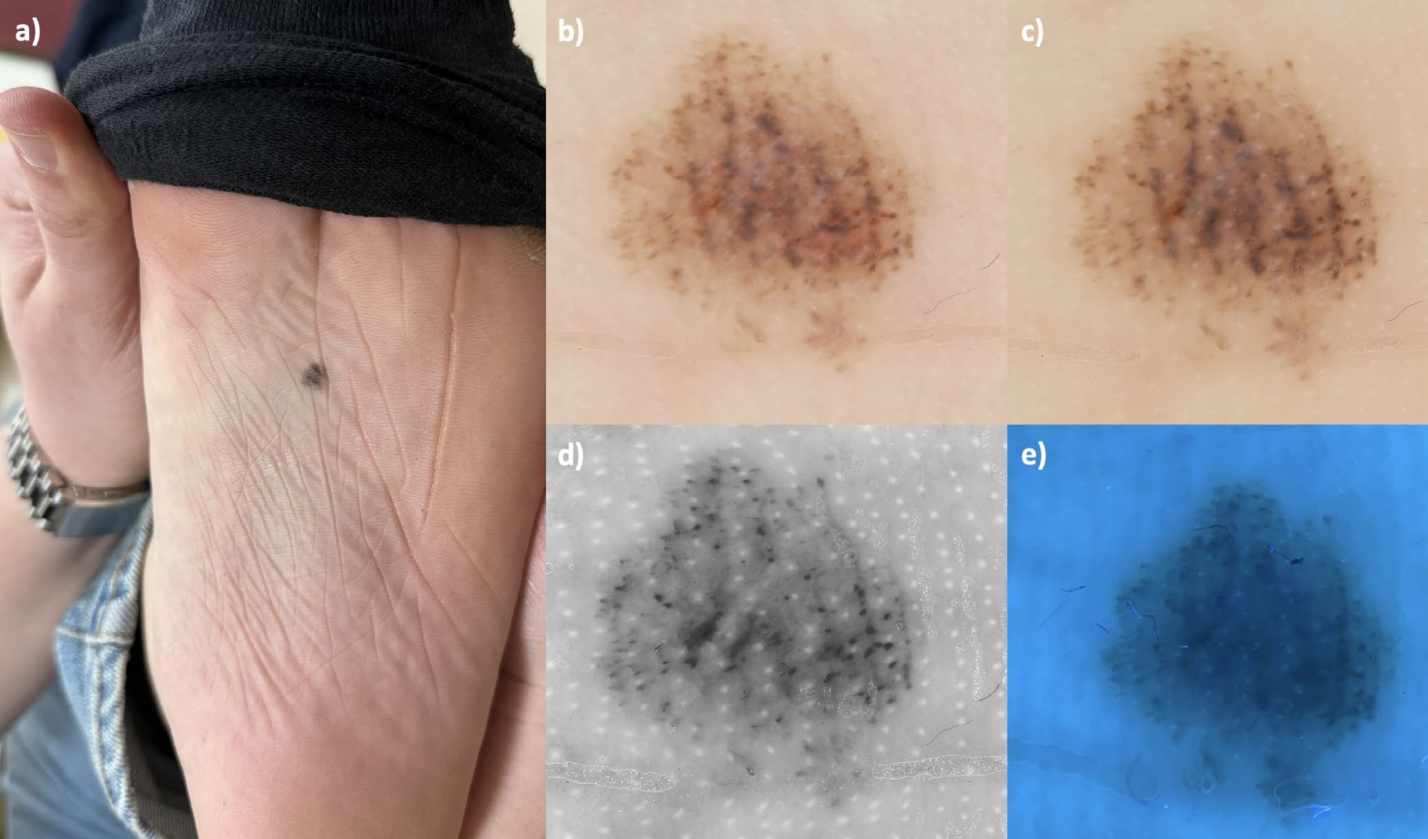
Acral nevus located on the left plantar surface. (a) Clinical image. (b) Polarized and (c) nonpolarized conventional dermoscopy show a parallel furrow pattern with globules. (d) Sub‐ultraviolet reflectance dermoscopy demonstrates the same pattern with markedly enhanced visualization of eccrine duct openings. (e) Ultraviolet‐induced fluorescence dermoscopy reveals similar overall morphology.

SUVRD demonstrated superiority in visualizing eccrine duct openings compared with CD (*p* = 0.008) and outperformed UVFD for both PFP and dots/clods (*p* = 0.047 and *p* = 0.036, respectively) (Figure 1).

#### 3.3.2 MIS

CD provided superior visualization of the PRP (*p* = 0.025) and dots/clods (*p* = 0.041) relative to UVFD.

**FIGURE 2:**
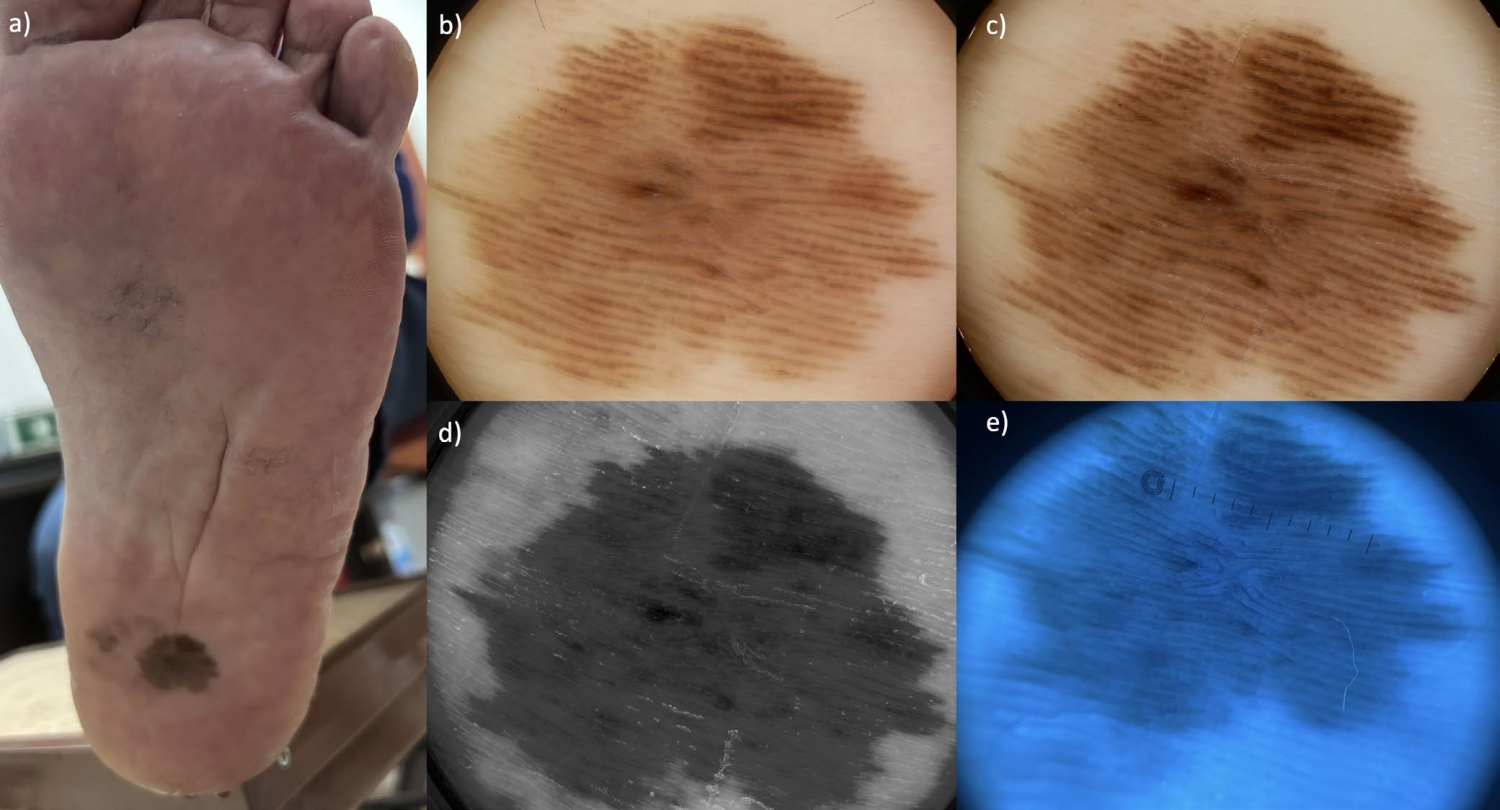
Melanoma in situ on the left heel. (a) Clinical image. (b) Polarized and (c) nonpolarized conventional dermoscopy reveal a parallel ridge pattern. (d) Sub‐ultraviolet reflectance dermoscopy demonstrates the same ridge‐dominant configuration with clear lesion margins. (e) Under ultraviolet‐induced fluorescence dermoscopy, however, the ridges appear lighter and furrows darker, creating a “reversed” pattern that may mimic a parallel furrow configuration. This optical inversion can obscure the true ridge pattern in melanoma.

SUVRD was more effective than UVFD in visualizing eccrine duct openings (*p* = 0.01) (Figure 2).

#### 3.3.3 Invasive Melanoma

CD distinctly outperformed UVFD for the PRP, radial lines, blue–white structureless areas, and blotches (*p* < 0.001, *p* = 0.028, *p* = 0.002, and *p* = 0.015, respectively) in invasive cases.

**FIGURE 3:**
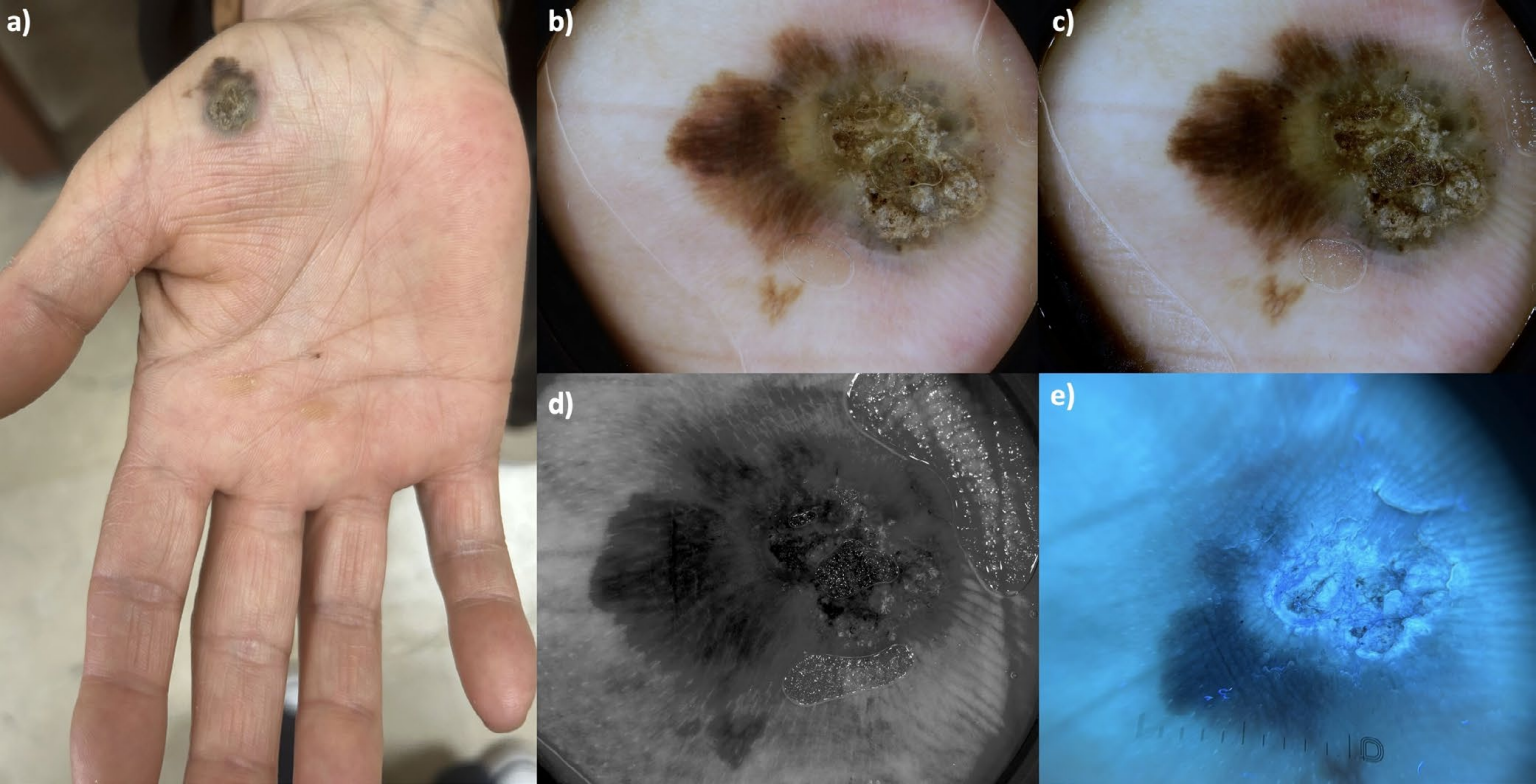
Invasive melanoma located on the right palm. (a) Clinical image. (b) Polarized and (c) nonpolarized conventional dermoscopy reveal a parallel ridge pattern with radial lines, blue–white structureless areas, scaling, and ulceration. (d) Sub‐ultraviolet reflectance dermoscopy shows a less distinct ridge pattern, with loss of the blue–white structureless area, but the eccrine duct openings are more clearly visible. (e) Ultraviolet‐induced fluorescence dermoscopy demonstrates further attenuation of structural detail, with both the ridge pattern and blue–white areas poorly defined and eccrine duct openings indistinct.

SUVRD surpassed UVFD for the PRP and radial lines (*p* = 0.007 and *p* = 0.028, respectively) (Figure 3).

Together, these findings highlight CD as superior for visualizing key melanoma‐specific criteria, while sUVRD provides valuable complementary information, especially for eccrine duct openings and selected benign features. UVFD, although associated with lower overall feature detectability, may still offer adjunctive value when interpreted alongside CD and sUVRD.

Table 2 presents the dermoscopic features of the diagnostic subgroups. Post hoc pairwise comparisons of these features, performed using Tukey's test, are summarized in Tables S3 and S4.

**TABLE 2:** Evaluation of dermoscopic features using various imaging devices in nevi, melanoma in situ, and invasive melanoma.

Dermoscopic findings	Dermoscopic imaging device			*p*	Post hoc Tukey's test
	CD score Mean (SD)	sUVRD score Mean (SD)	UVFD score Mean (SD)		
Nevi *n* = 22					
Eccrine duct opening	2.82 ± 1.62 (*n* = 22)	3.95 ± 1.33 (*n* = 22)	2.98 ± 1.62 (*n* = 14)	**0.007**	sUVRD>CD
Borders	4.77 ± 0.53 (*n* = 22)	4.86 ± 0.47 (*n* = 22)	4.48 ± 0.76 (*n* = 14)	0.062	—
Parellel furrow pattern	4.85 ± 0.56 (*n* = 13)	4.77 ± 0.59 (*n* = 13)	4.00 ± 0.82 (*n* = 7)	**0.019**	CD, sUVRD>UVFD
Globules/dots	4.55 ± 0.69 (*n* = 11)	4.36 ± 1.03 (*n* = 11)	3.12 ± 1.21 (*n* = 7)	**0.014**	CD, sUVRD>UVFD
Fibrillar pattern	4.67 ± 0.82 (*n* = 6)	4.17 ± 0.98 (*n* = 6)	3.13 ± 1.48 (*n* = 5)	**0.01**	CD>UVFD
Parellel ridge pattern	5.00 ± 0 (*n* = 4)	4.50 ± 1.00 (*n* = 4)	3.50 ± 0.71 (*n* = 2)	0.147	—
Melanoma in situ, *n* = 12					
Eccrine duct opening	2.75 ± 1.29 (*n* = 12)	3.58 ± 1.31 (*n* = 12)	2.01 ± 1.25 (*n* = 10)	**0.012**	sUVRD>UVFD
Borders	4.92 ± 0.29 (*n* = 12)	5.00 ± 0 (*n* = 12)	4.80 ± 0.42 (*n* = 10)	0.282	—
Parellel ridge pattern	4.75 ± 0.46 (*n* = 8)	3.50 ± 0.93 (*n* = 8)	3.00 ± 1.67 (*n* = 6)	**0.016**	CD>UVFD
Globules/dots	4.75 ± 0.50 (*n* = 4)	2.50 ± 1.91 (*n* = 4)	2.09 ± 1.15 (*n* = 3)	**0.029**	CD>UVFD
Blotch	4.75 ± 0.50 (*n* = 4)	4.75 ± 0.50 (*n* = 4)	3.72 ± 1.15 (*n* = 3)	0.082	—
Invasive melanoma, *n* = 8					
Eccrine duct opening	3.12 ± 1.46 (*n* = 8)	3.88 ± 1.46 (*n* = 8)	2.72 ± 1.63 (*n* = 6)	0.201	—
Borders	4.62 ± 0.52 (*n* = 8)	4.38 ± 1.19 (*n* = 8)	4.50 ± 0.55 (*n* = 6)	0.837	—
Parellel ridge pattern	4.86 ± 0.38 (*n* = 7)	4.29 ± 0.76 (*n* = 7)	3.01 ± 0.63 (*n* = 6)	**< 0.001**	CD, sUVRD>UVFD
Blue–white structureless areas	4.86 ± 0.38 (*n* = 7)	2.14 ± 1.46 (*n* = 7)	2.00 ± 1.00 (*n* = 5)	**< 0.001**	CD>sUVRD, UVFD
Ulceration	4.71 ± 0.76 (*n* = 7)	4.29 ± 1.11 (*n* = 7)	4.36 ± 0.82 (*n* = 6)	0.594	—
Scale	4.80 ± 0.45 (*n* = 5)	4.80 ± 0.45 (*n* = 5)	4.27 ± 0.96 (*n* = 4)	0.309	—
Radial lines/streaks	5.00 ± 0 (*n* = 4)	5.00 ± 0 (*n* = 4)	3.33 ± 1.15 (*n* = 3)	**0.013**	CD, sUVRD>UVFD
Blotch	5.00 ± 0 (*n* = 4)	4.00 ± 0.82 (*n* = 4)	2.10 ± 1.41 (*n* = 2)	**0.013**	CD>UVFD

*Note:* Dermoscopic findings that could not be evaluated on any device were excluded from *n*. Bold values indicate statistically significant associations (*p* 〈 0.05).

Abbreviations: CD: conventional dermoscopy, sUVRD: sub‐ultraviolet reflectance dermoscopy, UVFD: ultraviolet‐induced fluorescence dermoscopy, SD: standard deviation.

## 4 Discussion

Acral melanocytic nevi typically present as junctional or compound melanocytic proliferations, characterized by relatively small, symmetric, and well‐circumscribed lentiginous and nested melanocytic growths along the dermal–epidermal junction. These architectural features correspond well with dermoscopic observations: the parallel furrow, lattice‐like, and fibrillar patterns. In particular, the PFP reflects melanocytic nests located within the crista profunda limitans beneath surface furrows, forming melanin columns in the stratum corneum that appear as linear pigmentation on dermoscopy. Likewise, the fibrillar pattern arises from oblique melanin alignment in the stratum corneum, induced by mechanical pressure on weight‐bearing sites. These clinicopathological correlations explain the consistency with which benign nevus patterns were detected across all three imaging modalities in our study.

Acral melanoma is strongly associated with the PRP, characterized dermoscopically by band‐like brown‐to‐black pigmentation along surface ridges. Histopathologically, this corresponds to solitary, atypical melanocytes proliferating preferentially within the crista profunda intermedia. Thus, while nevi and melanomas may both present as pigmented acral lesions, their dermoscopic hallmarks are based on fundamentally different histological architectures, reinforcing the diagnostic value of pattern recognition [9, 10].

The depth of optical penetration in dermoscopy is strongly wavelength‐dependent [11]. CD employs visible light, which penetrates relatively deeply into the dermis, allowing visualization of both epidermal and superficial dermal structures. In contrast, UV‐based techniques utilize shorter wavelengths within the UVA (320–400 nm) and sub‐ultraviolet or violet–blue (400–425 nm) spectrum. These wavelengths exhibit shallower penetration due to increased absorption and scattering by epidermal chromophores, particularly melanin and keratin.

Previous studies have emphasized the capability of sUVRD in highlighting pigment distribution according to its depth. Minagawa et al. demonstrated that pigment networks in melanocytic nevi remain clearly visible or even enhanced under sUVRD, whereas malignant melanomas often show reduced pigment network visibility due to deeper melanin localization [3]. Similarly, Caposiena Caro et al. reported that blue nevi, characterized by deeply located melanocytes, became less conspicuous with sUVRD [12]. Akay et al. observed that Spitz nevi displaying structureless dark brown or black areas on CD revealed a dense, well‐organized pigment network under sUVRD [13]. This difference is likely attributable to the abundant melanin in the stratum corneum and the horizontal arrangement of melanocytes, characteristic of the fascicular growth pattern observed in Reed nevi. SUVRD accentuates these features due to its preferential interaction with superficial melanin.

In melanomas, when the pigment network resides primarily at the epidermal basal layer, it is often clearly visible under sUVRD. It is plausible that microinvasion or increased Breslow thickness may alter optical reflectance due to atypical melanocytes extending into the papillary dermis, leading to a slightly blurred or whitish‐grayish appearance. Therefore, sUVRD may not only assist in identifying the most suspicious area of a lesion for targeted biopsy but also offer a clue to tumor depth estimation; however, further studies with larger cohorts are needed to confirm this potential association.

Previous reports support the ability of sUVRD to enhance visualization of several dermoscopic features, including ulceration, crusts, comedo‐like openings, milia‐like cysts, multiple aggregated yellow (MAY) globules, fissures, ridges and vascular morphology [8, 14]. Building on these observations, a notable finding of our study was the superior visualization of eccrine duct openings with sUVRD compared with both CD and UVFD. This observation can be attributed to the specific optical behavior of sub‐ultraviolet light in the acral epidermis. At this wavelength, there is enhanced contrast between the keratin‐rich stratum corneum of the ridges and the eccrine ostia, where the architecture is interrupted and the optical properties are altered by the presence of eccrine ducts and sweat. The 405 nm illumination may accentuate subtle differences in absorption and backscattering at these transition points, making the eccrine openings appear as better‐defined negative or refractive structures against the surrounding epidermis. From a practical perspective, improved detection of eccrine ostia with sUVRD may assist in evaluating acral patterns, particularly when distinguishing parallel furrow or fibrillar configurations from ridge‐dominant patterns; however, these dermoscopic patterns are not invariably diagnostic, and overlap can occur in a subset of acral melanomas.

Ulceration represents another dermoscopic parameter for which UV‐based modalities have been suggested to provide potential advantages. UVFD has been reported to enhance visualization of relatively fresh erosions due to bilirubin‐related fluorescence in dried crusts, potentially aiding confirmation of ulceration in both melanoma and nonmelanoma skin cancers [2]. Similarly, sUVRD has been described as improving ulcer detection in pigmented BCCs and possibly contributing to diagnostic assessment in melanoma [3]. In our cohort, however, UV‐based modalities did not demonstrate a consistent advantage over CD in identifying ulceration. This discrepancy may relate to the limited sample size, acral localization, or lesion morphology, indicating that the added value of UV‐based modalities in AMLs remains to be clarified.

During image acquisition, we observed that the clarity of dermoscopic structures in sUVRD varied depending on surface conditions, such as dryness or hyperkeratosis. In our practical experience, insufficient hydration or imperfect contact may lead to uneven reflectance or localized image darkening, representing a potential technical consideration that can influence image quality in UV‐based modalities. Optimization of contact, including the use of immersion fluid or ultrasound gel, may help improve surface uniformity in future studies. In a case report evaluating malignant melanoma with sUVRD, areas of the heel with markedly thick stratum corneum appeared dark gray under violet illumination, potentially complicating lesion assessment [15]. We noted a similar appearance in some heel lesions. These observations highlight the importance of interpreting sUVRD in conjunction with CD, particularly in hyperkeratotic acral sites.

Another technical consideration emerged with UVFD. Yürekli et al. described that in normal acral skin, ridges appear lighter and grooves darker under UV illumination, a finding they termed the “highway view” as ridges resembled white parallel lines separated by darker furrows [16]. While this visualization may facilitate the distinction of normal dermatoglyphic structures, we observed that in some acral melanomas, the PRP appeared less distinct and occasionally resembled a PFP under UVFD. This finding was not statistically analyzed in our study but may represent a potential diagnostic pitfall that warrants further investigation.

Although we cannot confirm a direct causal relationship, these optical characteristics of UV‐dermoscopy may have contributed to the lower visibility scores observed for UVFD and sUVRD compared with CD in our series of AMLs. These observations should not be interpreted as universal limitations of UV‐based dermoscopy, but rather as modality‐specific characteristics that may influence image appearance.

Our study's limitations include the lack of histopathological confirmation for lesions classified as nevi. Nevi were included only if they exhibited a stable clinical course over a minimum follow‐up period of 5 years and demonstrated dermoscopic features consistent with well‐established benign acral patterns, with no clinical or dermoscopic signs of atypia. While this approach reflects routine clinical practice and ethical considerations in young patients with unequivocally benign‐appearing lesions, the lack of pathological verification inevitably introduces a residual risk of misclassification. Given the known clinical and dermoscopic overlap between benign nevi and very early‐stage melanoma, it is conceivable that a small proportion of lesions categorized as nevi may have represented early melanoma [17, 18]. This limitation may have influenced the interpretation of visibility scoring differences across dermoscopic modalities, particularly in subgroup analyses.

## 5 Conclusions

Based on our observations, CD remains the most reliable modality for visualizing melanoma‐specific features of AMLs, particularly the PRP and blue–white structureless areas.

UV‐based techniques, although not superior in overall visibility scores, may offer complementary information in selected cases when combined with CD.

While larger studies are required to substantiate these observations, we believe that integrating UV‐based modalities into routine dermoscopic evaluation could enhance lesion assessment and promote a more comprehensive understanding of acral morphology.

## Funding

The authors have nothing to report.

## Ethics Statement

This study was approved by the Clinical Research Ethics Committee of the Ankara University Faculty of Medicine (Approval No: I05‐424‐25).

## Consent

Consent for the publication of recognizable patient photographs or other identifiable material was obtained by the authors and included at the time of article submission to the journal stating that all patients gave consent with the understanding that this information may be publicly available.

## Conflicts of Interest

The authors declare no conflicts of interest.

## Supporting information


**Table S1:** Epidemiological features of the patients with acral melanocytic lesions.
**Table S2:** Clinical features of the acral melanocytic lesions.
**Table S3:** Post hoc pairwise comparisons (Tukey's test) of dermoscopic features presented in Table 1.
**Table S4:** Post hoc pairwise comparisons (Tukey's test) of dermoscopic features presented in Table 2.

## Data Availability

The data that support the findings of this study are available from the corresponding author upon reasonable request.
