# Supplementary material for: Acral Melanocytic Lesions Under Conventional, Sub‐Ultraviolet Reflectance, and Ultraviolet‐Induced Fluorescence Dermoscopy: A Comparative Analysis
Source: Int J Dermatol. 2026 Mar 16;65(8):1625–32. doi: 10.1111/ijd.70384 (PMC13342755; doi:10.1111/ijd.70384)
Supplement: Supplementary file 1 — Table S1: Epidemiological features of the patients with acral melanocytic lesions. Table S2: Clinical features of the acral melanocytic lesions. Table S3: Post hoc pairwise comparisons (Tukey's test) of dermoscopic features presented in Table 1. Table S4: Post hoc pairwise comparisons (Tukey's test) of dermoscopic features presented in Table 2. [file IJD-65-1625-s001.docx]

**Supplementary Materials**

**Table S1. Epidemiological features of the patients with acral melanocytic lesions**

|  | **Nevi**  **(22 lesions from 17 patients)** | **Melanoma in situ**  **(12 lesions from 12 patients)** | **Invasive melanoma**  **(8 lesions from 8 patients)** | **p** |
| --- | --- | --- | --- | --- |
| Gender (male/female) | 3/14 | 4/8 | 2/6 | 0.703 |
| Mean age (SD), years | 27.2 (15.1) | 66.5 (15.1) | 65.0 (20.3) | **<0.001^a^** |

Abbreviation: SD: standard deviation

**^a^**As a result of the Tukey post-hoc test, the mean age of patients with invasive melanoma and melanoma in situ was statistically significantly higher than that of patients with nevi (both p < 0.001).

**Table S2. Clinical features of the acral melanocytic lesions**

|  | **Nevi**  **(22 lesions from 17 patients)** | **Melanoma in situ**  **(12 lesions from 12 patients)** | **Invasive melanoma**  **(8 lesions from 8 patients)** |
| --- | --- | --- | --- |
|  | **Lesions, n (%)** | | |
| Location (Hand)   - Palm - Thumb - Middle finger - Index finger   Location (Foot)   - Sole - Lateral side - Medial side - Lesser toe | 3 (13.6)  0 (0)  0 (0)  0 (0)  16 (72.7)  2 (9.1)  0 (0)  1 (4.5) | 0 (0)  2 (16.7)  1 (8.3)  2 (16.7)  5 (41.7)  1 (8.3)  1 (8.3)  0 (0) | 1 (12.5)  0 (0)  0 (0)  0 (0)  6 (75.0)  0 (0)  1 (12.5)  0 (0) |
| Skin phototype   - 1 - 2 - 3 - 4 | 0 (0)  14 (63.6)  6 (27.3)  2 (9.1) | 0 (0)  4 (33.3)  7 (58.3)  1 (8.3) | 1 (12.5)  3 (37.5)  3 (37.5)  1 (12.5) |

**Table S3.** **Post-hoc pairwise comparisons (Tukey’s test) of dermoscopic features presented in Table 1.**

| **Dermoscopic findings** | **Groups** | **Mean difference** | **Std. Error** | **p** |
| --- | --- | --- | --- | --- |
| Eccrine duct opening | CD – sUVRD | -0.976 | 0.248 | **< 0.001** |
|  | CD – UVFD | 0.251 | 0.279 | 0.642 |
|  | sUVRD – UVFD | 1.227 | 0.279 | **< 0.001** |
| Parellel ridge pattern | CD – sUVRD | 0.842 | 0.271 | **0.011** |
|  | CD – UVFD | 1.774 | 0.279 | **< 0.001** |
|  | sUVRD – UVFD | 0.931 | 0.279 | **0.009** |
| Globules/dots | CD – sUVRD | 0.688 | 0.366 | 0.166 |
|  | CD – UVFD | 1.616 | 0.416 | **0.002** |
|  | sUVRD – UVFD | 0.928 | 0.416 | 0.084 |
| Parellel furrow pattern | CD – sUVRD | 0.067 | 0.251 | 0.962 |
|  | CD – UVFD | 0.978 | 0.295 | **0.008** |
|  | sUVRD – UVFD | 0.911 | 0.295 | **0.012** |
| Blotch | CD – sUVRD | 0.444 | 0.333 | 0.402 |
|  | CD – UVFD | 1.512 | 0.388 | **0.004** |
|  | sUVRD – UVFD | 1.068 | 0.388 | **0.038** |
| Radial lines/streaks | CD – sUVRD | 0.500 | 0.377 | 0.410 |
|  | CD – UVFD | 1.385 | 0.451 | **0.024** |
|  | sUVRD – UVFD | 0.885 | 0.451 | 0.164 |
| Blue-white structureless areas | CD – sUVRD | 2.714 | 0.562 | **0.002** |
|  | CD – UVFD | 2.857 | 0.631 | **0.002** |
|  | sUVRD – UVFD | 0.143 | 0.631 | 0.972 |
| Fibrillar pattern | CD – sUVRD | 0.500 | 0.365 | 0.395 |
|  | CD – UVFD | 1.530 | 0.392 | **0.009** |
|  | sUVRD – UVFD | 1.030 | 0.392 | 0.063 |

Abbreviations: CD: Conventional dermoscopy, sUVRD: Sub-ultraviolet reflectance dermoscopy, UVFD: Ultraviolet-induced fluorescence

Bold values indicate statistically significant associations (p < 0.05).

**Table S4. Post-hoc pairwise comparisons (Tukey’s test) of dermoscopic features presented in Table 2.**

| **Dermoscopic findings** | **Groups** | **Mean difference** | **Std. Error** | **p** |
| --- | --- | --- | --- | --- |
| Nevi - Eccrine duct opening | CD – sUVRD | -1.136 | 0.356 | **0.008** |
|  | CD – UVFD | -0.158 | 0.418 | 0.920 |
|  | sUVRD – UVFD | 0.978 | 0.418 | 0.063 |
| Nevi - Parellel furrow pattern | CD – sUVRD | 0.077 | 0.248 | 0.949 |
|  | CD – UVFD | 0.846 | 0.304 | **0.027** |
|  | sUVRD – UVFD | 0.769 | 0.304 | **0.047** |
| Nevi - Globules/dots | CD – sUVRD | 0.182 | 0.395 | 0.891 |
|  | CD – UVFD | 1.428 | 0.460 | **0.016** |
|  | sUVRD – UVFD | 1.247 | 0.46 | **0.036** |
| Nevi - Fibrillar pattern | CD – sUVRD | 0.500 | 0.365 | 0.395 |
|  | CD – UVFD | 1.530 | 0.392 | **0.009** |
|  | sUVRD – UVFD | 1.030 | 0.392 | 0.063 |
| MIS - Eccrine duct opening | CD – sUVRD | -0.833 | 0.453 | 0.183 |
|  | CD – UVFD | 0.743 | 0.482 | 0.292 |
|  | sUVRD – UVFD | 1.577 | 0.482 | 0.01 |
| MIS - Parellel ridge pattern | CD – sUVRD | 1.250 | 0.532 | 0.086 |
|  | CD – UVFD | 1.750 | 0.584 | **0.025** |
|  | sUVRD – UVFD | 0.500 | 0.584 | 0.676 |
| MIS - Globules/dots | CD – sUVRD | 2.250 | 0.696 | 0.051 |
|  | CD – UVFD | 2.655 | 0.786 | **0.041** |
|  | sUVRD – UVFD | 0.405 | 0.786 | 0.867 |
| IM - Parellel ridge pattern | CD – sUVRD | 0.571 | 0.317 | 0.213 |
|  | CD – UVFD | 1.850 | 0.33 | **< 0.001** |
|  | sUVRD – UVFD | 1.279 | 0.334 | **0.007** |
| IM - Blue-white structureless areas | CD – sUVRD | 2.714 | 0.562 | **0.002** |
|  | CD – UVFD | 2.857 | 0.631 | **0.002** |
|  | sUVRD – UVFD | 0.143 | 0.631 | 0.972 |
| IM - Radial lines/streaks | CD – sUVRD | 0.000 | 0.408 | 1.000 |
|  | CD – UVFD | 1.670 | 0.459 | **0.028** |
|  | sUVRD – UVFD | 1.670 | 0.459 | **0.028** |
| IM - Blotch | CD – sUVRD | 1.000 | 0.457 | 0.187 |
|  | CD – UVFD | 2.900 | 0.629 | **0.015** |
|  | sUVRD – UVFD | 1.900 | 0.629 | 0.068 |

Abbreviations: MIS: Melanoma in situ, IM: Invasive melanoma, CD: Conventional dermoscopy, sUVRD: Sub-ultraviolet reflectance dermoscopy, UVFD: Ultraviolet-induced fluorescence

Bold values indicate statistically significant associations (p < 0.05).
